# Designing equitable workplace dietary interventions: perceptions of intervention deliverers

**DOI:** 10.1186/s12889-017-4810-x

**Published:** 2017-10-16

**Authors:** Sarah A. Smith, Shelina Visram, Claire O’Malley, Carolyn Summerbell, Vera Araujo-Soares, Frances Hillier-Brown, Amelia A. Lake

**Affiliations:** 1 Fuse, UKCRC Centre for Translational Research in Public Health, Newcastle, UK; 2 School of Medicine, Pharmacy and Health, Durham University Queen’s Campus, Stockton-On-Tees, UK; 3 School of Applied Social Sciences, Durham University, Durham, UK; 4 Institute of Health and Society, Newcastle University, Newcastle-upon-Tyne, UK; 5 Department of Science, School of Science, Engineering and Design, Teesside University, Middlesbrough, UK

**Keywords:** Workplace health, Obesity, Intervention, Diet, Inequalities

## Abstract

**Background:**

Workplaces are a good setting for interventions that aim to support workers in achieving a healthier diet and body weight. However, little is known about the factors that impact on the feasibility and implementation of these interventions, and how these might vary by type of workplace and type of worker. The aim of this study was to explore the views of those involved in commissioning and delivering the Better Health at Work Award, an established and evidence-based workplace health improvement programme.

**Methods:**

One-to-one semi-structured interviews were conducted with 11 individuals in North East England who had some level of responsibility for delivering workplace dietary interventions. Interviews were transcribed verbatim and analysed using thematic framework analysis.

**Results:**

A number of factors were felt to promote the feasibility and implementation of interventions. These included interventions that were cost-neutral (to employee and employer), unstructured, involved colleagues for support, took place at lunchtimes, and were well-advertised and communicated via a variety of media. Offering incentives, not necessarily monetary, was perceived to increase recruitment rates. Factors that militate against feasibility and implementation of interventions included worksites that were large in size and remote, working patterns including shifts and working outside of normal working hours that were not conducive to workers being able to access intervention sessions, workplaces without appropriate provision for healthy food on site, and a lack of support from management.

**Conclusions:**

Intervention deliverers perceived that workplace dietary interventions should be equally and easily accessible (in terms of cost and timing of sessions) for all staff, regardless of their job role. Additional effort should be taken to ensure those staff working outside normal working hours, and those working off-site, can easily engage with any intervention, to avoid the risk of intervention-generated inequalities (IGIs).

**Electronic supplementary material:**

The online version of this article (10.1186/s12889-017-4810-x) contains supplementary material, which is available to authorized users.

## Background

Recent worldwide estimates show that 13% of adults (11% of men and 15% of women) were obese in 2014, and 39% of adults (38% of men and 40% of women) were overweight [1]. In the UK, 26.0% men and 23.8% women were obese in 2013 [2]. It is widely acknowledged that there is a global need to develop and evaluate dietary interventions conducted in various settings to address this growing problem [3, 4]. The workplace has the potential to be such a setting, providing an ideal environment for health interventions to tackle dietary behaviours [5, 6]. Interventions in workplace settings can impact on a large proportion of the adult population as those in employment can spend up to two-thirds of their day at work [7–11]. Studies that assess the effectiveness of interventions in reducing inequalities in obesity are needed, with a focus particularly on macro- or organisation-level interventions that have the potential to address the entire gradient [12]. Studies that have focussed on environmental changes and education have been shown to have positive short term effects on dietary intakes of participants’ [13–15]. However, there is a lack of evidence of UK-based workplace intervention studies that focus on the practicalities and implications when designing and implementing an intervention within the workplace setting. Research exists that provides an overview of organisational workplace interventions, however there is limited information on practice or implementation. Furthermore, there is a need to evaluate any differential impacts of interventions by socio-economic status [16, 17]. The Better Health at Work Award (BHWA) is an established and evidence-based workplace health improvement programme, with a high coverage (21.4%) of the working-age population employed in the North East of England [18]. BHWA is a partnership between the 12 Local Authorities in the region, the Northern Trade Unions Congress (TUC) and the National Health Service (NHS), and was developed ‘to give recognition and endorsement to those organisations that are committed to developing a sustainable culture of health and wellbeing in the workplace’ [19, 20]. The BHWA gives support to workplaces and staff to offer the chance to be fitter, healthier and safer, and is free to all organisations, across sectors, of any size in the region. It involves over 400 employers and is therefore a potentially valuable resource to acquire knowledge of workplace interventions.

Although the focus of the BHWA is determined by the needs and preferences of individual workplaces, most include advice and support in relation to healthy eating (see Additional file 1 for examples). These interventions were not evaluated and this was attributed to lack of funding and capacity for evaluation. The aim of this research was to explore the perceptions and experiences of commissioners and deliverers of the BHWA in terms of designing and implementing dietary interventions within workplace settings. The intention was to identify the components of successful interventions, in order to inform the development and piloting of an intervention in a local workplace.

## Methods

A pragmatic qualitative approach, involving one-to-one semi-structured interviews with a sample of BHWA stakeholders, was employed to meet the above aim. This provided a framework for comparison between interviews, as well as allowing participants to raise additional issues. The BHWA has a number of stakeholders involved in delivering the scheme, see Table 1. A convenience sampling approach was employed; although it entails a risk of bias, this approach is commonly used in exploratory and service development research [21]. The study was designed to inform future intervention development and evaluation, rather than test or build theory.

**Table 1:** Breakdown of the BHWA stakeholders’ role

Health Improvement Commissioners
Health Improvement Commissioners, employed by Local Authorities in the region, have knowledge in terms of funding BHWA, and have a broad knowledge of organisations in the region delivering dietary interventions.
Health Leads
Health Leads are working closely with organisations to deliver BHWA so have knowledge of the barriers organisations have come across and how they have overcome these. The local Health Leads help to train Health Advocates within the workforce.
Health Advocates
Health Advocates are employees of the workplaces signed up to BHWA and bring knowledge of the complexity of workplaces that needs to be taken in to account when designing and delivering an intervention in the workplace setting.

An invitation email was sent by Northern TUC to Health Leads and Health Advocates within workplaces that have conducted a diet- or nutrition-related intervention (in the broadest sense). It is not possible to know exact numbers of health leads and advocates approached to take part. However, an estimated 118 organisations participated in BHWA in the financial year 2014–15 and there were multiple health leads and advocates per organisation. An email was also sent to those who had been involved in commissioning the BHWA (*n* = 12) within local authority public health teams across the North East. Individuals were invited to contact the researcher directly if they were interested in taking part. From those who responded to the initial invitation (*n* = 14), three did not respond to follow-up emails.

The final sample included three local authority commissioners of the BHWA, alongside two Health Leads and six Health Advocates within organisations who were part of the BHWA scheme. Interviews were conducted between June and August 2015 by telephone and digitally recorded, before being anonymised and transcribed verbatim. The mean length of the interviews was 23 min (range 9–38 min). One interviewer (SS), who has extensive experience of qualitative research, conducted all interviews. The interviewer and participants did not know one another; apart from an email to arrange the interview, no previous contact had been made.

Interviews were structured to identify respondents’ experiences of commissioning, designing and/or implementing dietary interventions in workplace settings, and to identify facilitators and barriers to successful intervention delivery in these settings. Topic guides were written and reviewed by the authors. The topic guides were not pre-tested; however, after the first two interviews it was clear that minimal adaptations were needed and the guides were deemed fit for purpose after minor changes were made. These changes included putting less emphasis on the interviewee’s role, and ascertaining whether there were on-site catering facilities at their workplace.

### Data analysis

The interview data were analysed by the lead author (SAS), with a second researcher (COM) independently analysing a sample of four transcripts. The transcripts were analysed using a combination of thematic and framework analysis; the latter involves a systematic approach to qualitative data analysis to reduce researcher bias and increase the reliability of the analysis [22]. Analysis took place manually to ensure the researchers’ continued immersion in the data. Transcripts were divided into sections and arranged into themes and sub-themes by the researchers. The researchers’ analyses were compared to establish reliability with agreement on themes emerging from the data. There were no disagreements between the two researchers. A number of techniques to enhance rigour and reduce bias were used, these include: audio-recording interviews and note-taking; interviews were transcribed verbatim; the data and analyses were shared with and between colleagues; maintaining a reflexive approach throughout the fieldwork. No new themes were identified after interview nine, which fits with previous research that thematic data saturation is normally reached at 10–30 in depth interviews [23] therefore we are confident that saturation was reached.

## Results

**Fig. 1:**
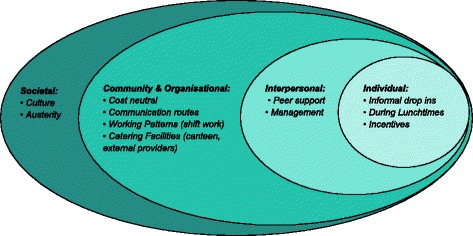
Socio-Ecological Model (SEM) for Workplace Dietary Behaviours

This analysis adapted the Social-Ecological Model (SEM) [24] of health promotion to a four-level ecological model designed to better understand workplace dietary behaviours, see Fig. 1. The model considers the interplay between individual, interpersonal, community, and societal factors and aims to guide intervention development targeting dietary behaviours in workplaces. During analysis, the emerging themes and subthemes were organised using Socio-Ecological Model, which has been used successfully in previous studies in health research [25, 26]. Findings from the interviews were grouped into these four levels influencing dietary behaviour, which were subdivided into specific factors.

### Participant characteristics

Participants were from a range of workplaces with a wide geographical spread across the North East of England. Job roles included health and safety officer, health trainer, medical professionals (such as a qualified nurse), administrators and public health commissioners. Six interviewees were public sector workers employed in local government agencies, while five worked for private industries.

### Individual factors

Interventions involving delivery of health messages in an unstructured manner (rather than structured content and having to attend every week) were perceived to be successful. Furthermore, if sessions were conducted at lunchtimes, attendance was reported to be good as staff were not required to use flexi-time. Offering incentives was also felt to enhance uptake, although, interestingly, non-monetary incentives, such as stress balls, free swimming or gym sessions, were perceived as most popular.

*‘Freebies. People love something free, even if it’s a stress ball they’ll turn up… it’s not the monetary value, it’s just to have something tangible’ *(Health Advocate 7)

### Interpersonal factors

#### Peer support

Interventions involving attendance with other colleagues for peer support were perceived to be successful and encouraged individuals to take part in initiatives. In one organisation, short weight management sessions were delivered during lunchtimes and were received positively, as illustrated by the following quote:

*‘people enjoyed the 10 minutes with each other, you know, supporting each other so that was really good’*(Health Advocate 3)

#### Management

The importance of the involvement of management was highlighted. If managers were supportive, in terms of staff accessing initiatives, this was perceived as having a positive impact on uptake and retention, and the converse was also true. Increasing knowledge amongst management of the positive relationship between investing in employee health and reduction in absenteeism rates, and subsequent increases in productivity, was thought to be beneficial.

*‘So sometimes it is just finding some sort of carrot, and that usually does relate to the chief exec. You know, “The chief exec has asked for this or he really supports this”, and that helps bring people on board.’* (Health Advocate 3)

However, sometimes management was felt to represent a barrier that could not be overcome, particularly in the case of changes in company ownership, which often brought a cultural conflict in views on health, as illustrated by the quote below.

*‘it was a different management style and a different plant director, he didn’t promote health at all. He thought … if you had anything wrong with you that you had to go out and pay for it and the company weren’t going to fund anything… everything was taken away and even the stuff from the canteen, like the subsidised, that was all removed, and it was like, “go and look after yourself”’* (Health Advocate 2)

### Community and organisational influences

#### Cost

At the time of conducting the research, workplaces were being stretched and workplace initiatives targeting dietary behaviour were perceived to be less of a priority as a result. Participants reported that canteens were being closed, and healthy food provision was no longer seen as important. To incentivise management to invest in employee health, participants felt that interventions must be cost-neutral.

*‘on the whole most organisations are open to simple things that they can do. You know. If they can do it simply and it’s going to be cost-effective and it’s going to be minimal to them, you know what I mean, in terms of cost then they’re going to employ it’* (Health Advocate 7)

#### Communication routes

Offering interventions at the workplace was in itself seen as a facilitator to uptake and completion as people were easily contactable on site. Using the various communications channels available in a workplace, such as email, staff intranet, and posters in the canteen, was perceived to be useful in recruitment and retention. Emails that prompted and encouraged staff to continue were seen as particularly useful.

*‘if it’s delivered in your organisation, it’s easier because you’ve got peoples emails to hand, they’re on a directory and things like that, so facilitating that, that’s one side that’s easier’* (Health Lead 2)

#### Working patterns

Participants reported that there were particular groups within the workforce that may be at risk of missing out on initiatives and healthier food provision. These included shift workers and truck drivers who reportedly experienced barriers to taking part in initiatives due to working antisocial hours, often during the night, when most initiatives were delivered during the daytime.

*‘A lot of shift workers there, and of course that sort of thing is a barrier because the award generally only happens during the day…. (the award) didn’t stop them getting the takeaways at one o’ clock in the morning, but that’s just the way it is’* (Health Lead 2)

The type of work was felt to impact on participation in interventions; for example, chemical sites have designated areas for eating that must be adhered to, so initiatives were restricted to reception areas, the canteen, or occupational health venues. Not all staff accessed these facilities and therefore some were described as missing out on the opportunity to take part. Furthermore, chemical and engineering plants are often very large in size, which can be a barrier to initiatives reaching staff across the site.

*‘it depends on the kind of facility we’re working in. I mean obviously if you’ve got a steel foundry you’re not going to be able to do much in there, particularly if they haven’t got a canteen…’* (Health Lead 2)

#### Catering facilities

It became apparent through the interviews that some workplaces had little or no food provision on site and limited access to healthy food; for example, vending machines as the only option. Due to the lack of food provision, employees were relying on alternative sources, such as external food outlets and mobile caterers. Often the food options available for purchase were unhealthy in content and size. Shift workers reportedly tend to obtain food offsite from takeaway facilities and mobile caterers and therefore miss out on onsite food provision, for example, healthier canteen options.

*‘We’ve lost what used to be the canteen. It was really, really good … We have the sandwich man, as we call him. Well his food’s pretty good but, you know, he brings big double-sized buns in instead of single-sized buns. He brings too much of a selection of chocolate when he hardly sells any but it’s all in front of you. You know, he never brings any fruit in. He brings the odd case of yoghurts in, but it’s generally all stodge’* (Health Advocate 1)

### Societal influences

There were broader societal factors identified that influence workplaces in terms of encouraging healthy eating and making healthier dietary choices obtainable.

#### Culture

Amongst employees, there was a sense coming from management that the workplace was solely a place of work, and that this impacted on the likelihood of employees engaging with initiatives.

*‘At the end of the day these people are in work and these people work to make a profit for their employer…. So actually releasing people [to take part in activities] can be quite difficult’* (Health Lead 1)

This ‘work’place culture became more problematic when coupled with companies being target-driven, as these targets tended to take priority over staff health and wellbeing. Target driven workplaces were industries that employed a range of workers, including white collar (general office workers, administrative); blue collar (manual work); and grey collar (principally white collar but perform blue collar tasks such as skilled technicians, engineers). Subsequently an increasing proportion of participants were reported to be working through lunch without eating at all.

*‘Targets were the main issue. And I had to give up the healthy living group while we were really target-driven. I think it’s just people’s workloads’* (Health Advocate 10)

There was a clear association with management and workforce participation in workplace initiatives, but there was also the wider influence of societal attitudes towards work, particularly in relation to differences between the private and public sectors. Although seen in the private sector, there was perceived to be a greater feeling of being conflicted amongst the workforce in public sector roles to participate in health initiatives. Public sector workers were doubly conflicted; firstly, taking time away from work to attend the initiatives; and secondly that the work they were taking time from was publicly funded.

*‘it’s trying to convince them to take part, but then again it all comes back to the funding, because they’re funded by public money’* (Health Advocate 3)

#### Austerity

The study took place during a time of austerity, which was perceived to have an impact on workplace health due to reported cutbacks in the provision of healthy food, not least the closure of canteens. Participants described how the workforce were feeling the economic situation and opting for cheaper alternatives that were often higher in calorie content and poor nutritional content.

*‘I do know some places that are in the middle of nowhere and the only thing that comes round is a van. Now while they may do something like a cottage pie or a baked potato, other than that it’s burgers and really high fat greasy foods which are not particularly good, but it’s what people want because it’s the quick fix’* (Health Lead 2)

## Discussion

It is recognised that the workplace is a good setting in which to deliver health-promoting activities. In this study, dietary workplace initiatives perceived as being successful were those that were delivered in an unstructured capacity (rather than structured content and having to attend every week), at convenient times, and involved colleagues in providing support to one another. Initiatives being well-advertised and communicated via different avenues, as well as offering an incentive, were also reported to be facilitators to recruitment and retention.

This study has identified several inter-linked factors within workplace settings that influence dietary behaviours. Our model suggests that workplaces under financial pressure (austerity) may result in a management decision to close onsite catering and canteens. Without an onsite price-competitive canteen the workforce often relies heavily on external sources of catering and food provision whilst at work. Food provided by these external sources, such as takeaways and food outlets that pitch nearby or on site (the ‘sandwich man’ or ‘van’) was reported to be of poor nutritional quality, and served in large quantities.

Furthermore, the findings from this study highlight the possibility that workplace dietary interventions could contribute to inequalities by benefitting those less-disadvantaged (Intervention-Generated Inequalities or IGIs) [12]. This study has identified that shift workers are reportedly disadvantaged due to working antisocial hours, often during the night, when most initiatives were delivered during the daytime. Coupled with the remoteness of some sites, the large size of sites, the nature of the work conducted, and closure of canteens, shift workers cannot access healthier options that other members of the same workplace can.

It is clear from the findings of this study in relation to the SEM and the emerging workplace IGIs, that multi-component ecological interventions are required that address the wider context rather than individual behaviour change interventions that can exacerbate inequalities.

**Fig. 2:**
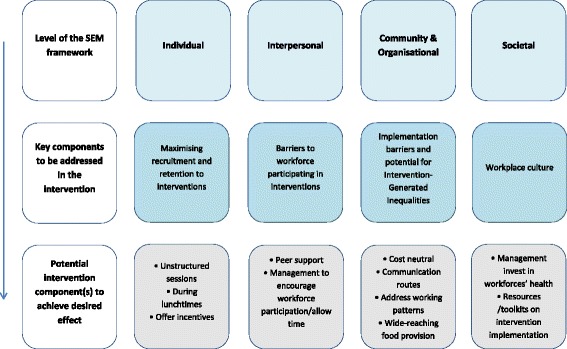
Key components identified as feasible to implement when developing future workplace interventions

Lorenc [27] found that structural workplace interventions, provision of resources and fiscal interventions such as tobacco pricing showed some evidence of reducing inequalities. A review by Hillier-Brown et al. [16] also showed that ‘upstream’ preventative interventions are less likely to increase health inequalities than ‘downstream’ interventions. The components identified from this work that are feasible to implement when developing future workplace interventions are outlined in Fig. 2.

A key finding from this study is that in order to be able to develop and deploy a workplace intervention it is crucial to actively involve those responsible for management. Only with their involvement can interventions be successfully implemented and barriers to participation eliminated. Employees feeling able to and comfortable with attending initiatives ‘guilt-free’ hinged on management’s attitude towards them taking time away from work to take part. Linnan et al. [28] found that few managers (41%) agreed that employers have a responsibility to encourage employees to make healthy lifestyle choices. There was disbelief that investing in the workforce in terms of health would see an improvement in recruitment, retention and productivity. The study went on to show that managers considered the main barriers to implementing initiatives to be lack of employee time to participate, lack of staff time, production conflicts, and cost of offering the programme. It is important that managers understand the short- and longer-term outcomes of unhealthy diets and what their business can stand to gain from interventions targeting healthier eating behaviours. Shaping management outcome expectations and beliefs that an intervention can be successful with their support is paramount [29]. Intervention deliverers will stand to gain if time and resources are invested in shaping management beliefs and expectations before an intervention is implemented. Profit-making is essential to successful business, therefore, to appeal to management, interventions must be designed to be simple to implement and cost-effective or indeed cost-neutral. Cost analysis of interventions is limited and would be useful for employers’ in informing what type of intervention is feasible, in both the short (implementation) and long term (maintenance). For example, the cost of implementing an environmental intervention has been shown to be marginal compared to nutrition education [30].

Workplace culture needs to be considered when designing and implementing future workplace interventions and the impact management can have on recruitment and retention. Fitzgerald et al. [31] found that employees appreciated the investment employers made in the intervention and were reassured that their employer concerns were not just about profit-making. Crump et al. [32] found that support from management improved employee participation but only in certain subgroups of the workforce, with blue-collar workers more likely to be influenced by management support than white collar workers. Linnan et al. [28] demonstrated that ‘different levels of managers vary in their beliefs’ with regards to health promotion at work. Even in large organisations the role of a single individual can be crucial in shaping what is on offer. Previous research on how to implement change can potentially support future endeavours that would need to first target these individuals in order to then be able to bring about change. Training is required at a high level so that health-promoting messages can be cascaded throughout the workforce. Guidance, in particular toolkits around how best to deliver an intervention in the workplace, is needed.

The strengths of this study are that participants were from a range of workplaces covering a wide geographical spread across the North East of England. Limitations of this study include the small sample size, convenience sampling approach and associated risks of bias, despite every attempt being made to recruit additional participants to the study. Perhaps the study involving being interviewed and asked to comment on current employment roles was off-putting. Another limitation is that the interview participants were predominantly from large engineering and chemical processing sites that employ hundreds of staff. There was no representation from small businesses in the study. This is interesting in itself, that uptake of small businesses to the BHWA award was low. In addition, this could highlight that working for a small business may be a contributing factor to lack of access to healthy initiatives in the workplace. Although enlightening in terms of revealing barriers to intervention design and delivery in large worksites, and the impact of shift work, the sample is not representative of the wider North East of England working demographic.

## Conclusions

Our findings indicate that multi-component ecological interventions are required that address the wider context of workplace health and avoid exacerbating inequalities. The role of management and the impact of austerity on local food provision in particular need to be considered. Intervention deliverers viewed that workplace dietary interventions should be equally and easily accessible (in terms of timing of sessions and cost) for all staff, regardless of their role. Additional effort should be taken to ensure those staff working outside standard working hours, and those working off-site, can easily engage with any intervention, to avoid the risk of intervention-generated inequalities.

## Additional file

Additional file 1: Box 1. Examples of existing BHWA workplace interventions as identified from interviews. A list of examples of existing BHWA workplace interventions. (DOCX 13 kb)

## Abbreviations

BHWA: Better health at work award
Fuse: The Centre for Translational Research in Public Health is a UK Clinical Research Collaboration (UKCRC) Public Health Research Centre of Excellence
IGI’s: Intervention-Generated Inequalities
MRC: Medical Research Council
NHS: National Health Service
SEM: Socio-ecological model
TUC: Trades Unions Congress
UK: United Kingdom
UKCRC: UK Clinical Research Collaboration
WHO: World Health Organisation
