# Supplementary material for: Designing equitable workplace dietary interventions: perceptions of intervention deliverers
Source: BMC Public Health. 2017 Oct 16;17:808. doi: 10.1186/s12889-017-4810-x (PMC5644102; doi:10.1186/s12889-017-4810-x)
Supplement: Additional file 1: Box 1. — Examples of existing BHWA workplace interventions as identified from interviews. A list of examples of existing BHWA workplace interventions. (DOCX 13 kb) [file 12889_2017_4810_MOESM1_ESM.docx]

**Additional File 1**

**Box 1: Examples of existing BHWA workplace interventions as identified from interviews**

**Category 1: Provision of healthier free foods:** free fruit days, healthy food tasting sessions

**Category 2: Changes to the canteen environment:** traffic light system on products, healthy salad bars, posters in the canteen promoting healthier options, provision of healthier products in vending machines

**Category 3: Providing tools to aid dietary change:** healthy eating plate, food diaries, leaflets/booklets (on portion control, salt intake, grains, how to read food labels), provision of healthy recipe files

**Category 4: Electronic resources:** directed to websites providing information on healthy eating, staff emails received with information on healthier options and lifestyles, staff intranet has healthy recipes and campaigns, electronic information booklets

**Category 5: Free sessions aimed at dietary change:** talks on mood, food, healthy eating

**Category 6: Free sessions aimed at weight loss/management:** unstructured weight loss and weight management groups, provision of free vouchers for Slimming World
